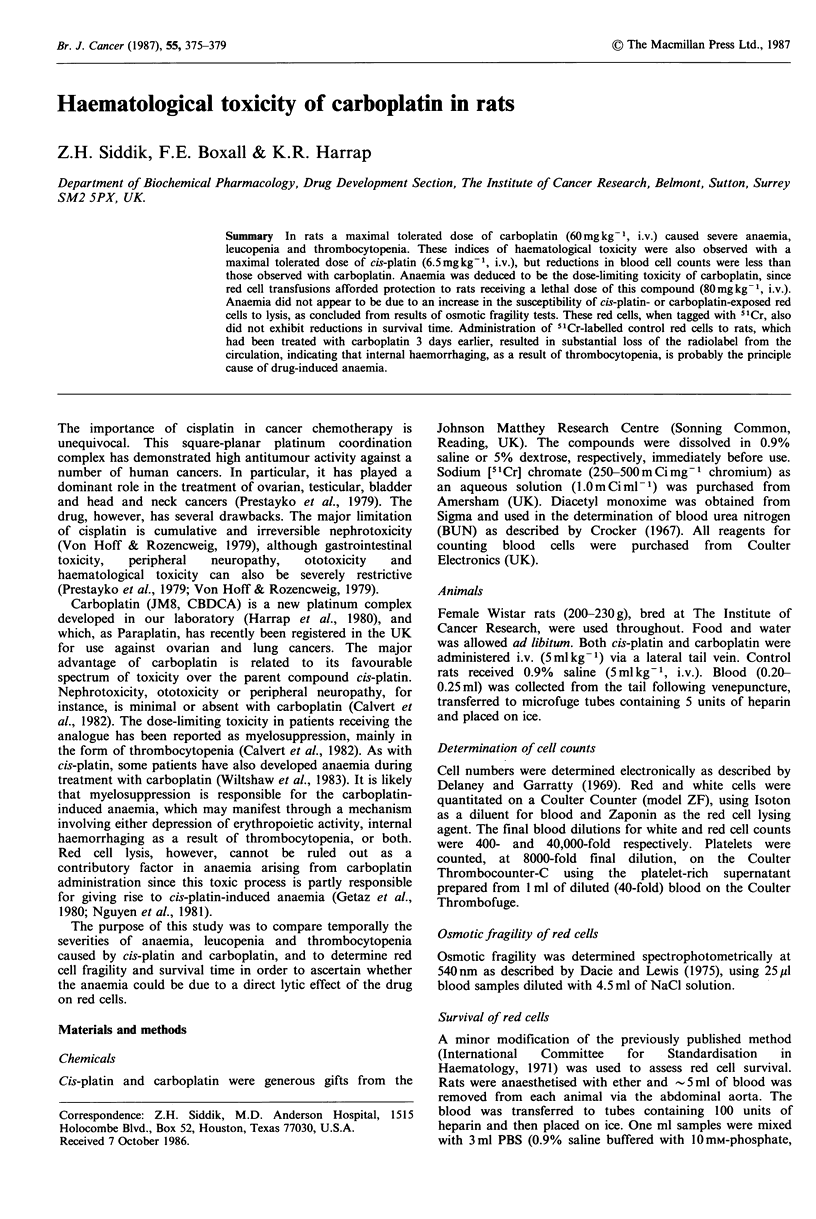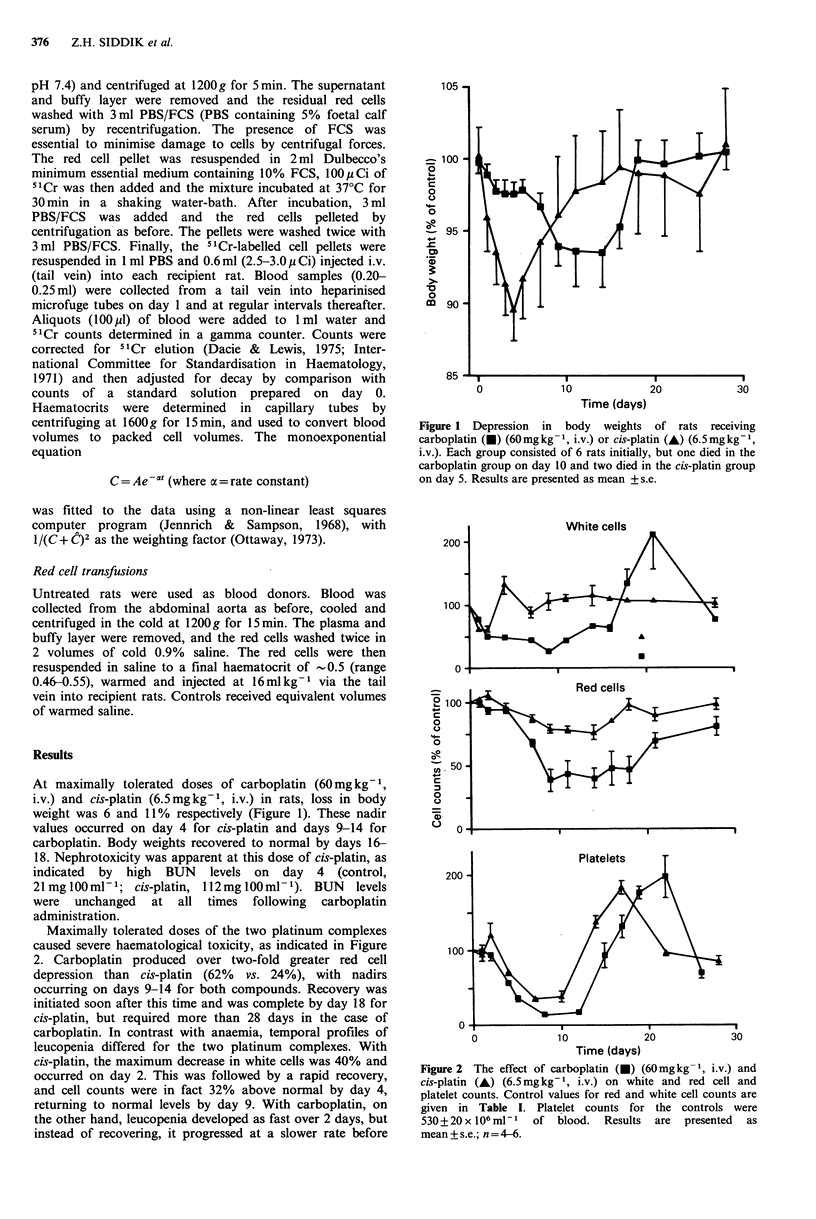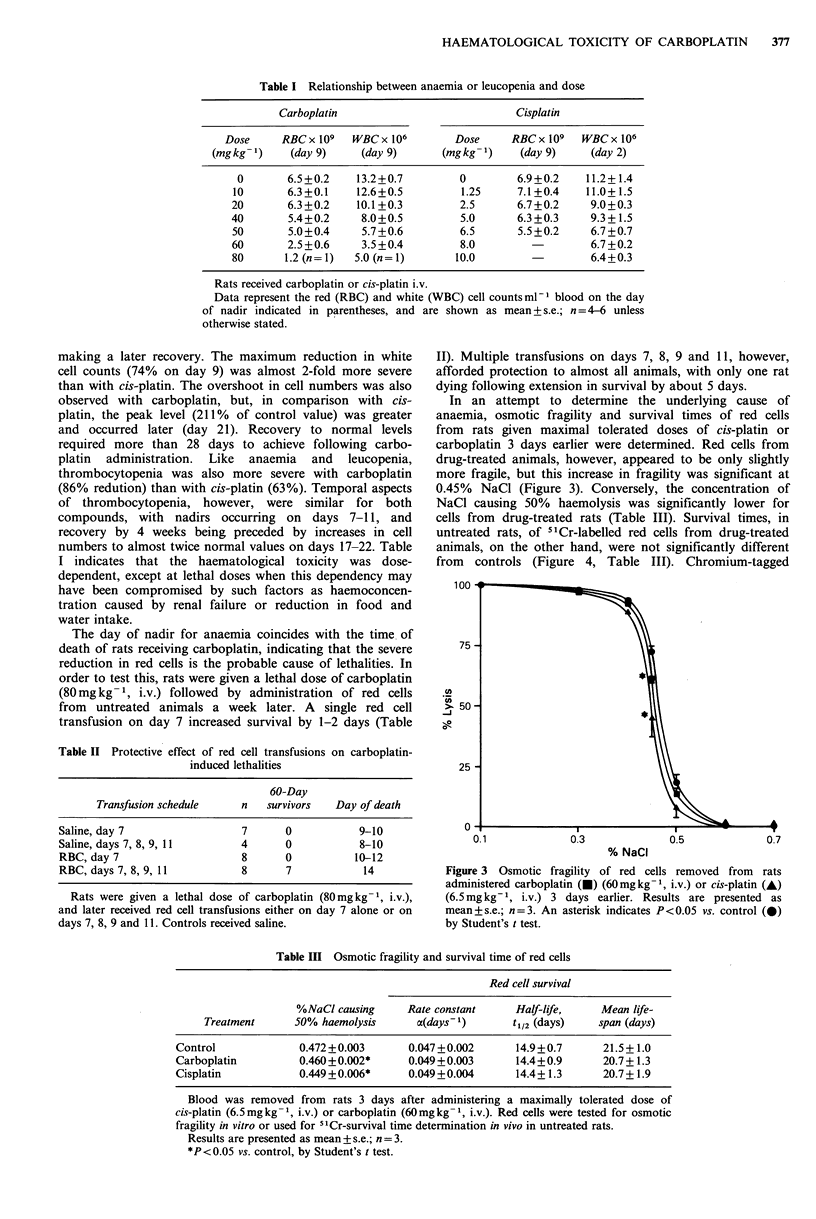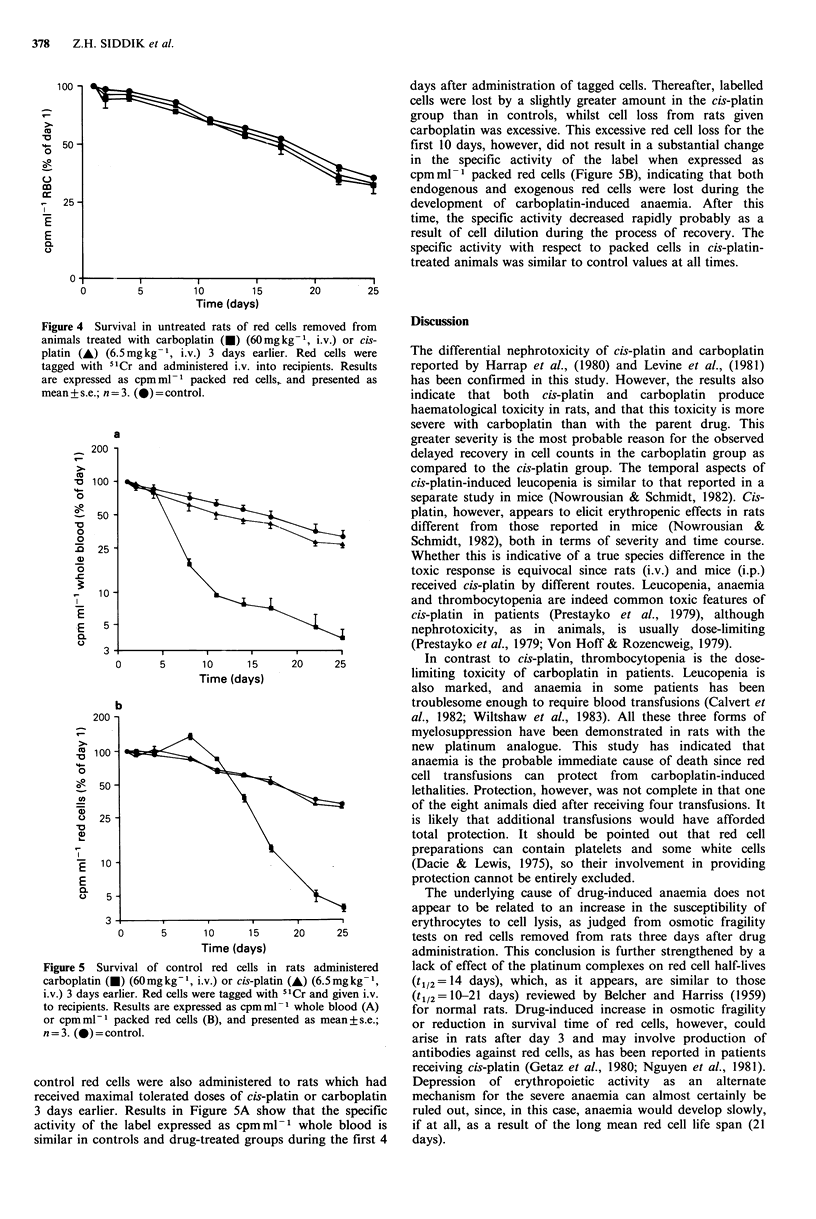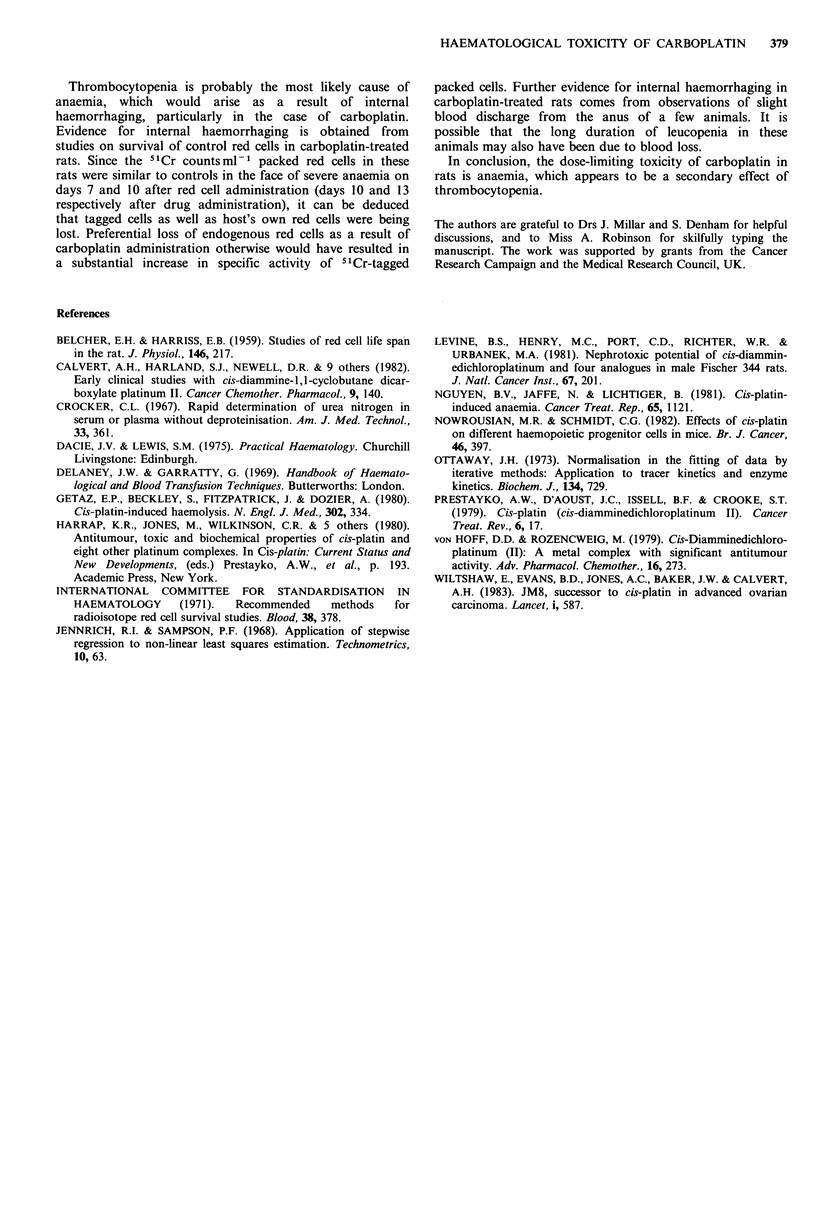# Haematological toxicity of carboplatin in rats.

**DOI:** 10.1038/bjc.1987.75

**Published:** 1987-04

**Authors:** Z. H. Siddik, F. E. Boxall, K. R. Harrap

## Abstract

In rats a maximal tolerated dose of carboplatin (60 mg kg-1, i.v.) caused severe anaemia, leucopenia and thrombocytopenia. These indices of haematological toxicity were also observed with a maximal tolerated dose of cis-platin (6.5 mg kg-1, i.v.), but reductions in blood cell counts were less than those observed with carboplatin. Anaemia was deduced to be the dose-limiting toxicity of carboplatin, since red cell transfusions afforded protection to rats receiving a lethal dose of this compound (80 mg kg-1, i.v.). Anaemia did not appear to be due to an increase in the susceptibility of cis-platin- or carboplatin-exposed red cells to lysis, as concluded from results of osmotic fragility tests. These red cells, when tagged with 51Cr, also did not exhibit reductions in survival time. Administration of 51Cr-labelled control red cells to rats, which had been treated with carboplatin 3 days earlier, resulted in substantial loss of the radiolabel from the circulation, indicating that internal haemorrhaging, as a result of thrombocytopenia, is probably the principle cause of drug-induced anaemia.


Br. J. Cancer (1987), 55, 375–379 © The Macmillan Press Ltd., 1987

### Haematological toxicity of carboplatin in rats

Z.H. Siddik, F.E. Boxall & K.R. Harrap

*Department of Biochemical Pharmacology, Drug Development Section, The Institute of Cancer Research, Belmont, Sutton, Surrey SM2 5PX, UK.*

**Summary** In rats a maximal tolerated dose of carboplatin ($60 mg kg^{-1}$, i.v.) caused severe anaemia, leucopenia and thrombocytopenia. These indices of haematological toxicity were also observed with a maximal tolerated dose of *cis*-platin ($6.5 mg kg^{-1}$, i.v.), but reductions in blood cell counts were less than those observed with carboplatin. Anaemia was deduced to be the dose-limiting toxicity of carboplatin, since red cell transfusions afforded protection to rats receiving a lethal dose of this compound ($80 mg kg^{-1}$, i.v.). Anaemia did not appear to be due to an increase in the susceptibility of *cis*-platin- or carboplatin-exposed red cells to lysis, as concluded from results of osmotic fragility tests. These red cells, when tagged with $^{51}Cr$, also did not exhibit reductions in survival time. Administration of $^{51}Cr$-labelled control red cells to rats, which had been treated with carboplatin 3 days earlier, resulted in substantial loss of the radiolabel from the circulation, indicating that internal haemorrhaging, as a result of thrombocytopenia, is probably the principle cause of drug-induced anaemia.

The importance of cisplatin in cancer chemotherapy is unequivocal. This square-planar platinum coordination complex has demonstrated high antitumour activity against a number of human cancers. In particular, it has played a dominant role in the treatment of ovarian, testicular, bladder and head and neck cancers (Prestayko *et al.*, 1979). The drug, however, has several drawbacks. The major limitation of cisplatin is cumulative and irreversible nephrotoxicity (Von Hoff & Rozencweig, 1979), although gastrointestinal toxicity, peripheral neuropathy, ototoxicity and haematological toxicity can also be severely restrictive (Prestayko *et al.*, 1979; Von Hoff & Rozencweig, 1979).

Carboplatin (JM8, CBDCA) is a new platinum complex developed in our laboratory (Harrap *et al.*, 1980), and which, as Paraplatin, has recently been registered in the UK for use against ovarian and lung cancers. The major advantage of carboplatin is related to its favourable spectrum of toxicity over the parent compound *cis*-platin. Nephrotoxicity, ototoxicity or peripheral neuropathy, for instance, is minimal or absent with carboplatin (Calvert *et al.*, 1982). The dose-limiting toxicity in patients receiving the analogue has been reported as myelosuppression, mainly in the form of thrombocytopenia (Calvert *et al.*, 1982). As with *cis*-platin, some patients have also developed anaemia during treatment with carboplatin (Wiltshaw *et al.*, 1983). It is likely that myelosuppression is responsible for the carboplatin-induced anaemia, which may manifest through a mechanism involving either depression of erythropoietic activity, internal haemorrhaging as a result of thrombocytopenia, or both. Red cell lysis, however, cannot be ruled out as a contributory factor in anaemia arising from carboplatin administration since this toxic process is partly responsible for giving rise to *cis*-platin-induced anaemia (Getaz *et al.*, 1980; Nguyen *et al.*, 1981).

The purpose of this study was to compare temporally the severities of anaemia, leucopenia and thrombocytopenia caused by *cis*-platin and carboplatin, and to determine red cell fragility and survival time in order to ascertain whether the anaemia could be due to a direct lytic effect of the drug on red cells.

### Materials and methods

### Chemicals

*Cis*-platin and carboplatin were generous gifts from the Johnson Matthey Research Centre (Sonning Common, Reading, UK). The compounds were dissolved in 0.9% saline or 5% dextrose, respectively, immediately before use. Sodium [$^{51}Cr$] chromate ($250–500 mCi mg^{-1}$ chromium) as an aqueous solution ($1.0 mCi ml^{-1}$) was purchased from Amersham (UK). Diacetyl monoxime was obtained from Sigma and used in the determination of blood urea nitrogen (BUN) as described by Crocker (1967). All reagents for counting blood cells were purchased from Coulter Electronics (UK).

### Animals

Female Wistar rats (200–230 g), bred at The Institute of Cancer Research, were used throughout. Food and water was allowed *ad libitum*. Both *cis*-platin and carboplatin were administered i.v. ($5 ml kg^{-1}$) via a lateral tail vein. Control rats received 0.9% saline ($5 ml kg^{-1}$, i.v.). Blood (0.20–0.25 ml) was collected from the tail following venepuncture, transferred to microfuge tubes containing 5 units of heparin and placed on ice.

### Determination of cell counts

Cell numbers were determined electronically as described by Delaney and Garratty (1969). Red and white cells were quantitated on a Coulter Counter (model ZF), using Isoton as a diluent for blood and Zaponin as the red cell lysing agent. The final blood dilutions for white and red cell counts were 400- and 40,000-fold respectively. Platelets were counted, at 8000-fold final dilution, on the Coulter Thrombocounter-C using the platelet-rich supernatant prepared from 1 ml of diluted (40-fold) blood on the Coulter Thrombofuge.

### Osmotic fragility of red cells

Osmotic fragility was determined spectrophotometrically at 540 nm as described by Dacie and Lewis (1975), using 25 $\mu l$ blood samples diluted with 4.5 ml of NaCl solution.

### Survival of red cells

A minor modification of the previously published method (International Committee for Standardisation in Haematology, 1971) was used to assess red cell survival. Rats were anaesthetised with ether and ~5 ml of blood was removed from each animal via the abdominal aorta. The blood was transferred to tubes containing 100 units of heparin and then placed on ice. One ml samples were mixed with 3 ml PBS (0.9% saline buffered with 10 mM-phosphate,

Correspondence: Z.H. Siddik, M.D. Anderson Hospital, 1515 Holocombe Blvd., Box 52, Houston, Texas 77030, U.S.A.
Received 7 October 1986.

pH 7.4) and centrifuged at 1200*g* for 5 min. The supernatant and buffy layer were removed and the residual red cells washed with 3 ml PBS/FCS (PBS containing 5% foetal calf serum) by recentrifugation. The presence of FCS was essential to minimise damage to cells by centrifugal forces. The red cell pellet was resuspended in 2 ml Dulbecco's minimum essential medium containing 10% FCS, 100 $\mu$Ci of $^{51}$Cr was then added and the mixture incubated at 37°C for 30 min in a shaking water-bath. After incubation, 3 ml PBS/FCS was added and the red cells pelleted by centrifugation as before. The pellets were washed twice with 3 ml PBS/FCS. Finally, the $^{51}$Cr-labelled cell pellets were resuspended in 1 ml PBS and 0.6 ml (2.5–3.0 $\mu$Ci) injected i.v. (tail vein) into each recipient rat. Blood samples (0.20–0.25 ml) were collected from a tail vein into heparinised microfuge tubes on day 1 and at regular intervals thereafter. Aliquots (100 $\mu$l) of blood were added to 1 ml water and $^{51}$Cr counts determined in a gamma counter. Counts were corrected for $^{51}$Cr elution (Dacie & Lewis, 1975; International Committee for Standardisation in Haematology, 1971) and then adjusted for decay by comparison with counts of a standard solution prepared on day 0. Haematocrits were determined in capillary tubes by centrifuging at 1600*g* for 15 min, and used to convert blood volumes to packed cell volumes. The monoexponential equation

$$C = Ae^{-\alpha t} \text{ (where } \alpha = \text{rate constant)}$$

was fitted to the data using a non-linear least squares computer program (Jennrich & Sampson, 1968), with $1/(C+\hat{C})^2$ as the weighting factor (Ottaway, 1973).

*Red cell transfusions*

Untreated rats were used as blood donors. Blood was collected from the abdominal aorta as before, cooled and centrifuged in the cold at 1200*g* for 15 min. The plasma and buffy layer were removed, and the red cells washed twice in 2 volumes of cold 0.9% saline. The red cells were then resuspended in saline to a final haematocrit of ~0.5 (range 0.46–0.55), warmed and injected at 16 ml kg$^{-1}$ via the tail vein into recipient rats. Controls received equivalent volumes of warmed saline.

### Results

At maximally tolerated doses of carboplatin (60 mg kg$^{-1}$, i.v.) and *cis*-platin (6.5 mg kg$^{-1}$, i.v.) in rats, loss in body weight was 6 and 11% respectively (Figure 1). These nadir values occurred on day 4 for *cis*-platin and days 9–14 for carboplatin. Body weights recovered to normal by days 16–18. Nephrotoxicity was apparent at this dose of *cis*-platin, as indicated by high BUN levels on day 4 (control, 21 mg 100 ml$^{-1}$; *cis*-platin, 112 mg 100 ml$^{-1}$). BUN levels were unchanged at all times following carboplatin administration.

Maximally tolerated doses of the two platinum complexes caused severe haematological toxicity, as indicated in Figure 2. Carboplatin produced over two-fold greater red cell depression than *cis*-platin (62% *vs.* 24%), with nadirs occurring on days 9–14 for both compounds. Recovery was initiated soon after this time and was complete by day 18 for *cis*-platin, but required more than 28 days in the case of carboplatin. In contrast with anaemia, temporal profiles of leucopenia differed for the two platinum complexes. With *cis*-platin, the maximum decrease in white cells was 40% and occurred on day 2. This was followed by a rapid recovery, and cell counts were in fact 32% above normal by day 4, returning to normal levels by day 9. With carboplatin, on the other hand, leucopenia developed as fast over 2 days, but instead of recovering, it progressed at a slower rate before

**Figure 1** Depression in body weights of rats receiving carboplatin (■) (60 mg kg$^{-1}$, i.v.) or *cis*-platin (▲) (6.5 mg kg$^{-1}$, i.v.). Each group consisted of 6 rats initially, but one died in the carboplatin group on day 10 and two died in the *cis*-platin group on day 5. Results are presented as mean ± s.e.

**Figure 2** The effect of carboplatin (■) (60 mg kg$^{-1}$, i.v.) and *cis*-platin (▲) (6.5 mg kg$^{-1}$, i.v.) on white and red cell and platelet counts. Control values for red and white cell counts are given in **Table I**. Platelet counts for the controls were 530 ± 20 × 10$^{6}$ ml$^{-1}$ of blood. Results are presented as mean ± s.e.; $n$ = 4–6.

**Table I** Relationship between anaemia or leucopenia and dose

| *Carboplatin* | | | *Cisplatin* | | |
|---|---|---|---|---|---|
| *Dose* (*mg kg*$^{-1}$) | *RBC* × $10^9$ (*day* 9) | *WBC* × $10^6$ (*day* 9) | *Dose* (*mg kg*$^{-1}$) | *RBC* × $10^9$ (*day* 9) | *WBC* × $10^6$ (*day* 2) |
| 0 | 6.5±0.2 | 13.2±0.7 | 0 | 6.9±0.2 | 11.2±1.4 |
| 10 | 6.3±0.1 | 12.6±0.5 | 1.25 | 7.1±0.4 | 11.0±1.5 |
| 20 | 6.3±0.2 | 10.1±0.3 | 2.5 | 6.7±0.2 | 9.0±0.3 |
| 40 | 5.4±0.2 | 8.0±0.5 | 5.0 | 6.3±0.3 | 9.3±1.5 |
| 50 | 5.0±0.4 | 5.7±0.6 | 6.5 | 5.5±0.2 | 6.7±0.7 |
| 60 | 2.5±0.6 | 3.5±0.4 | 8.0 | — | 6.7±0.2 |
| 80 | 1.2 ($n$=1) | 5.0 ($n$=1) | 10.0 | — | 6.4±0.3 |

Rats received carboplatin or *cis*-platin i.v.

Data represent the red (RBC) and white (WBC) cell counts ml$^{-1}$ blood on the day of nadir indicated in parentheses, and are shown as mean±s.e.; $n$=4–6 unless otherwise stated.

making a later recovery. The maximum reduction in white cell counts (74% on day 9) was almost 2-fold more severe than with *cis*-platin. The overshoot in cell numbers was also observed with carboplatin, but, in comparison with *cis*-platin, the peak level (211% of control value) was greater and occurred later (day 21). Recovery to normal levels required more than 28 days to achieve following carboplatin administration. Like anaemia and leucopenia, thrombocytopenia was also more severe with carboplatin (86% redution) than with *cis*-platin (63%). Temporal aspects of thrombocytopenia, however, were similar for both compounds, with nadirs occurring on days 7–11, and recovery by 4 weeks being preceded by increases in cell numbers to almost twice normal values on days 17–22. Table I indicates that the haematological toxicity was dose-dependent, except at lethal doses when this dependency may have been compromised by such factors as haemoconcentration caused by renal failure or reduction in food and water intake.

The day of nadir for anaemia coincides with the time of death of rats receiving carboplatin, indicating that the severe reduction in red cells is the probable cause of lethalities. In order to test this, rats were given a lethal dose of carboplatin (80 mg kg$^{-1}$, i.v.) followed by administration of red cells from untreated animals a week later. A single red cell transfusion on day 7 increased survival by 1–2 days (Table II). Multiple transfusions on days 7, 8, 9 and 11, however, afforded protection to almost all animals, with only one rat dying following extension in survival by about 5 days.

**Table II** Protective effect of red cell transfusions on carboplatin-induced lethalities

| *Transfusion schedule* | *n* | *60-Day survivors* | *Day of death* |
|---|---|---|---|
| Saline, day 7 | 7 | 0 | 9–10 |
| Saline, days 7, 8, 9, 11 | 4 | 0 | 8–10 |
| RBC, day 7 | 8 | 0 | 10–12 |
| RBC, days 7, 8, 9, 11 | 8 | 7 | 14 |

Rats were given a lethal dose of carboplatin (80 mg kg$^{-1}$, i.v.), and later received red cell transfusions either on day 7 alone or on days 7, 8, 9 and 11. Controls received saline.

In an attempt to determine the underlying cause of anaemia, osmotic fragility and survival times of red cells from rats given maximal tolerated doses of *cis*-platin or carboplatin 3 days earlier were determined. Red cells from drug-treated animals, however, appeared to be only slightly more fragile, but this increase in fragility was significant at 0.45% NaCl (Figure 3). Conversely, the concentration of NaCl causing 50% haemolysis was significantly lower for cells from drug-treated rats (Table III). Survival times, in untreated rats, of $^{51}$Cr-labelled red cells from drug-treated animals, on the other hand, were not significantly different from controls (Figure 4, Table III). Chromium-tagged

**Figure 3** Osmotic fragility of red cells removed from rats administered carboplatin (■) (60 mg kg$^{-1}$, i.v.) or *cis*-platin (▲) (6.5 mg kg$^{-1}$, i.v.) 3 days earlier. Results are presented as mean±s.e.; $n$=3. An asterisk indicates $P$<0.05 *vs.* control (●) by Student's $t$ test.

**Table III** Osmotic fragility and survival time of red cells

| | | *Red cell survival* | | |
|---|---|---|---|---|
| *Treatment* | *%NaCl causing 50% haemolysis* | *Rate constant* $\alpha$(*days*$^{-1}$) | *Half-life,* $t_{1/2}$ (days) | *Mean life-span* (*days*) |
| Control | 0.472±0.003 | 0.047±0.002 | 14.9±0.7 | 21.5±1.0 |
| Carboplatin | 0.460±0.002* | 0.049±0.003 | 14.4±0.9 | 20.7±1.3 |
| Cisplatin | 0.449±0.006* | 0.049±0.004 | 14.4±1.3 | 20.7±1.9 |

Blood was removed from rats 3 days after administering a maximally tolerated dose of *cis*-platin (6.5 mg kg$^{-1}$, i.v.) or carboplatin (60 mg kg$^{-1}$, i.v.). Red cells were tested for osmotic fragility *in vitro* or used for $^{51}$Cr-survival time determination *in vivo* in untreated rats.

Results are presented as mean±s.e.; $n$=3.

*$P$<0.05 *vs.* control, by Student's $t$ test.

**Figure 4** Survival in untreated rats of red cells removed from animals treated with carboplatin (■) ($60\,mg\,kg^{-1}$, i.v.) or *cis*-platin (▲) ($6.5\,mg\,kg^{-1}$, i.v.) 3 days earlier. Red cells were tagged with $^{51}Cr$ and administered i.v. into recipients. Results are expressed as $cpm\,ml^{-1}$ packed red cells, and presented as mean ± s.e.; $n=3$. (●) = control.

**Figure 5** Survival of control red cells in rats administered carboplatin (■) ($60\,mg\,kg^{-1}$, i.v.) or *cis*-platin (▲) ($6.5\,mg\,kg^{-1}$, i.v.) 3 days earlier. Red cells were tagged with $^{51}Cr$ and given i.v. to recipients. Results are expressed as $cpm\,ml^{-1}$ whole blood (A) or $cpm\,ml^{-1}$ packed red cells (B), and presented as mean ± s.e.; $n=3$. (●) = control.

control red cells were also administered to rats which had received maximal tolerated doses of *cis*-platin or carboplatin 3 days earlier. Results in Figure 5A show that the specific activity of the label expressed as $cpm\,ml^{-1}$ whole blood is similar in controls and drug-treated groups during the first 4 days after administration of tagged cells. Thereafter, labelled cells were lost by a slightly greater amount in the *cis*-platin group than in controls, whilst cell loss from rats given carboplatin was excessive. This excessive red cell loss for the first 10 days, however, did not result in a substantial change in the specific activity of the label when expressed as $cpm\,ml^{-1}$ packed red cells (Figure 5B), indicating that both endogenous and exogenous red cells were lost during the development of carboplatin-induced anaemia. After this time, the specific activity decreased rapidly probably as a result of cell dilution during the process of recovery. The specific activity with respect to packed cells in *cis*-platin-treated animals was similar to control values at all times.

### Discussion

The differential nephrotoxicity of *cis*-platin and carboplatin reported by Harrap *et al.*, (1980) and Levine *et al.*, (1981) has been confirmed in this study. However, the results also indicate that both *cis*-platin and carboplatin produce haematological toxicity in rats, and that this toxicity is more severe with carboplatin than with the parent drug. This greater severity is the most probable reason for the observed delayed recovery in cell counts in the carboplatin group as compared to the *cis*-platin group. The temporal aspects of *cis*-platin-induced leucopenia is similar to that reported in a separate study in mice (Nowrousian & Schmidt, 1982). *Cis*-platin, however, appears to elicit erythropenic effects in rats different from those reported in mice (Nowrousian & Schmidt, 1982), both in terms of severity and time course. Whether this is indicative of a true species difference in the toxic response is equivocal since rats (i.v.) and mice (i.p.) received *cis*-platin by different routes. Leucopenia, anaemia and thrombocytopenia are indeed common toxic features of *cis*-platin in patients (Prestayko *et al.*, 1979), although nephrotoxicity, as in animals, is usually dose-limiting (Prestayko *et al.*, 1979; Von Hoff & Rozencweig, 1979).

In contrast to *cis*-platin, thrombocytopenia is the dose-limiting toxicity of carboplatin in patients. Leucopenia is also marked, and anaemia in some patients has been troublesome enough to require blood transfusions (Calvert *et al.*, 1982; Wiltshaw *et al.*, 1983). All these three forms of myelosuppression have been demonstrated in rats with the new platinum analogue. This study has indicated that anaemia is the probable immediate cause of death since red cell transfusions can protect from carboplatin-induced lethalities. Protection, however, was not complete in that one of the eight animals died after receiving four transfusions. It is likely that additional transfusions would have afforded total protection. It should be pointed out that red cell preparations can contain platelets and some white cells (Dacie & Lewis, 1975), so their involvement in providing protection cannot be entirely excluded.

The underlying cause of drug-induced anaemia does not appear to be related to an increase in the susceptibility of erythrocytes to cell lysis, as judged from osmotic fragility tests on red cells removed from rats three days after drug administration. This conclusion is further strengthened by a lack of effect of the platinum complexes on red cell half-lives ($t_{1/2}=14$ days), which, as it appears, are similar to those ($t_{1/2}=10$–$21$ days) reviewed by Belcher and Harriss (1959) for normal rats. Drug-induced increase in osmotic fragility or reduction in survival time of red cells, however, could arise in rats after day 3 and may involve production of antibodies against red cells, as has been reported in patients receiving *cis*-platin (Getaz *et al.*, 1980; Nguyen *et al.*, 1981). Depression of erythropoietic activity as an alternate mechanism for the severe anaemia can almost certainly be ruled out, since, in this case, anaemia would develop slowly, if at all, as a result of the long mean red cell life span (21 days).

Thrombocytopenia is probably the most likely cause of anaemia, which would arise as a result of internal haemorrhaging, particularly in the case of carboplatin. Evidence for internal haemorrhaging is obtained from studies on survival of control red cells in carboplatin-treated rats. Since the $^{51}Cr$ counts $ml^{-1}$ packed red cells in these rats were similar to controls in the face of severe anaemia on days 7 and 10 after red cell administration (days 10 and 13 respectively after drug administration), it can be deduced that tagged cells as well as host's own red cells were being lost. Preferential loss of endogenous red cells as a result of carboplatin administration otherwise would have resulted in a substantial increase in specific activity of $^{51}Cr$-tagged packed cells. Further evidence for internal haemorrhaging in carboplatin-treated rats comes from observations of slight blood discharge from the anus of a few animals. It is possible that the long duration of leucopenia in these animals may also have been due to blood loss.

In conclusion, the dose-limiting toxicity of carboplatin in rats is anaemia, which appears to be a secondary effect of thrombocytopenia.

The authors are grateful to Drs J. Millar and S. Denham for helpful discussions, and to Miss A. Robinson for skilfully typing the manuscript. The work was supported by grants from the Cancer Research Campaign and the Medical Research Council, UK.

**References**

BELCHER, E.H. & HARRISS, E.B. (1959). Studies of red cell life span in the rat. *J. Physiol.*, **146**, 217.

CALVERT, A.H., HARLAND, S.J., NEWELL, D.R. & 9 others (1982). Early clinical studies with *cis*-diammine-1,1-cyclobutane dicarboxylate platinum II. *Cancer Chemother. Pharmacol.*, **9**, 140.

CROCKER, C.L. (1967). Rapid determination of urea nitrogen in serum or plasma without deproteinisation. *Am. J. Med. Technol.*, **33**, 361.

DACIE, J.V. & LEWIS, S.M. (1975). *Practical Haematology*. Churchill Livingstone: Edinburgh.

DELANEY, J.W. & GARRATTY, G. (1969). *Handbook of Haematological and Blood Transfusion Techniques*. Butterworths: London.

GETAZ, E.P., BECKLEY, S., FITZPATRICK, J. & DOZIER, A. (1980). *Cis*-platin-induced haemolysis. *N. Engl. J. Med.*, **302**, 334.

HARRAP, K.R., JONES, M., WILKINSON, C.R. & 5 others (1980). Antitumour, toxic and biochemical properties of *cis*-platin and eight other platinum complexes. In Cis-*platin: Current Status and New Developments*, (eds.) Prestayko, A.W., *et al.*, p. 193. Academic Press, New York.

INTERNATIONAL COMMITTEE FOR STANDARDISATION IN HAEMATOLOGY (1971). Recommended methods for radioisotope red cell survival studies. *Blood*, **38**, 378.

JENNRICH, R.I. & SAMPSON, P.F. (1968). Application of stepwise regression to non-linear least squares estimation. *Technometrics*, **10**, 63.

LEVINE, B.S., HENRY, M.C., PORT, C.D., RICHTER, W.R. & URBANEK, M.A. (1981). Nephrotoxic potential of *cis*-diammin-edichloroplatinum and four analogues in male Fischer 344 rats. *J. Natl. Cancer Inst.*, **67**, 201.

NGUYEN, B.V., JAFFE, N. & LICHTIGER, B. (1981). *Cis*-platin-induced anaemia. *Cancer Treat. Rep.*, **65**, 1121.

NOWROUSIAN, M.R. & SCHMIDT, C.G. (1982). Effects of *cis*-platin on different haemopoietic progenitor cells in mice. *Br. J. Cancer*, **46**, 397.

OTTAWAY, J.H. (1973). Normalisation in the fitting of data by iterative methods: Application to tracer kinetics and enzyme kinetics. *Biochem. J.*, **134**, 729.

PRESTAYKO, A.W., D'AOUST, J.C., ISSELL, B.F. & CROOKE, S.T. (1979). *Cis*-platin (*cis*-diamminedichloroplatinum II). *Cancer Treat. Rev.*, **6**, 17.

VON HOFF, D.D. & ROZENCWEIG, M. (1979). *Cis*-Diamminedichloroplatinum (II): A metal complex with significant antitumour activity. *Adv. Pharmacol. Chemother.*, **16**, 273.

WILTSHAW, E., EVANS, B.D., JONES, A.C., BAKER, J.W. & CALVERT, A.H. (1983). JM8, successor to *cis*-platin in advanced ovarian carcinoma. *Lancet*, **i**, 587.